# Design of Alginate/Gelatin Hydrogels for Biomedical Applications: Fine-Tuning Osteogenesis in Dental Pulp Stem Cells While Preserving Other Cell Behaviors

**DOI:** 10.3390/biomedicines12071510

**Published:** 2024-07-08

**Authors:** Zied Ferjaoui, Roberto López-Muñoz, Soheil Akbari, Fatiha Chandad, Diego Mantovani, Mahmoud Rouabhia, Roberto D. Fanganiello

**Affiliations:** 1 Oral Ecology Research Group (GREB), Faculté de Médecine Dentaire, Université Laval, Québec City, QC G1V 0A6, Canada; fatiha.chandad@greb.ulaval.ca (F.C.); mahmoud.rouabhia@fmd.ulaval.ca (M.R.); robertofanganiello@gmail.com (R.D.F.); 2 Laboratory for Biomaterials and Bioengineering, (CRC-Tier I), Department of Min-Met-Materials Eng and Regenerative Medicine, CHU de Quebec, Laval University, Quebec City, QC G1V 0A6, Canada; jose-roberto.lopez-munoz.1@ulaval.ca (R.L.-M.); diego.mantovani@gmn.ulaval.ca (D.M.); 3 Département de Génie Chimique, Université Laval, Québec City, QC G1V 0A6, Canada; soheil.akbari.1@ulaval.ca

**Keywords:** alginate, gelatin, hydrogel, stiffness, DPSCs, osteogenic differentiation

## Abstract

Alginate/gelatin (Alg-Gel) hydrogels have been used experimentally, associated with mesenchymal stromal/stem cells (MSCs), to guide bone tissue formation. One of the main challenges for clinical application is optimizing Alg-Gel stiffness to guide osteogenesis. In this study, we investigated how Alg-Gel stiffness could modulate the dental pulp stem cell (DPSC) attachment, morphology, proliferation, and osteogenic differentiation, identifying the optimal conditions to uncouple osteogenesis from the other cell behaviors. An array of Alg-Gel hydrogels was prepared by casting different percentages of alginate and gelatin cross-linked with 2% CaCl_2_. We have selected two hydrogels: one with a stiffness of 11 ± 1 kPa, referred to as “low-stiffness hydrogel”, formed by 2% alginate and 8% gelatin, and the other with a stiffness of 55 ± 3 kPa, referred to as “high-stiffness hydrogel”, formed by 8% alginate and 12% gelatin. Hydrogel analyses showed that the average swelling rates were 20 ± 3% for the low-stiffness hydrogels and 35 ± 2% for the high-stiffness hydrogels. The degradation percentage was 47 ± 5% and 18 ± 2% for the low- and high-stiffness hydrogels, respectively. Both hydrogel types showed homogeneous surface shape and protein (Alg-Gel) interaction with CaCl_2_ as assessed by physicochemical characterization. Cell culture showed good adhesion of the DPSCs to the hydrogels and proliferation. Furthermore, better osteogenic activity, determined by ALP activity and ARS staining, was obtained with high-stiffness hydrogels (8% alginate and 12% gelatin). In summary, this study confirms the possibility of characterizing and optimizing the stiffness of Alg-Gel gel to guide osteogenesis in vitro without altering the other cellular properties of DPSCs.

## 1. Introduction

Mesenchymal stromal/stem cells (MSCs) hold great potential in regenerative medicine because they differentiate into various cell types, including osteoblasts, which are responsible for bone tissue formation and regeneration [1,2]. Bone regeneration requires osteogenic cells and a scaffold to support the cells forming bone tissue. Alginate and gelatin hydrogels have emerged as promising biomaterials for tissue engineering applications due to their biocompatibility and ability to promote cell adhesion and differentiation [3,4]. However, while several studies have shown the osteogenic potential of these hydrogels, few have examined the effect of hydrogel stiffness on MSC osteogenic differentiation. Furthermore, the most important challenge is to design hydrogels that selectively promote osteogenesis without adversely impacting other cell behaviors, such as MSC cell differentiation to non-osteogenic lineages. It also has been shown that the proliferation and osteogenic functions of the osteoblasts fluctuated with increasing gelatin concentration in gelatin/nano-hydroxyapatite microcapsules (nHA), suggesting that the hydrogel properties must be balanced to provide an effective 3D osteoconductive microcapsule [5]. The alginate (1%)/gelatin (2.5%)/nHA (0.5%) microcapsule with a compressive modulus of 0.19 MPa ± 0.02 revealed maximum performance for cell proliferation and function, indicating a potential microcapsule composition to prepare building blocks for modular bone tissue engineering [5]. In another study, the effects of substrate stiffness on the differentiation of hMSCs, derived from the adult human bone marrow into adipogenic and osteogenic cells, has shown that the adipogenic and osteogenic differentiation of hMSCs is likely to occur on a substrate with stiffness similar to that of their in vivo microenvironments [6]. In the same sense, Žigon-Branc et al. [7] evaluated how the stiffness of gelatin-based hydrogels (having a storage modulus of 538, 3584, or 7263 Pa) affects the proliferation and differentiation of microspheroids formed from telomerase-immortalized human adipose-derived stem cells (hASC/hTERT). They showed better cell differentiation with calcium deposits when using softer hydrogels, suggesting that these soft hydrogels promote osteogenesis. The authors concluded that the encapsulation of adipose-derived stem cell microspheroids in gelatin-based hydrogels has promising potential for future applications in bone regeneration. A cell-imprinted poly(dimethylsiloxane)/hydroxyapatite nanocomposite substrate was developed by Kamguyan et al. [8] Engaging topographical, mechanical, and chemical signals to stimulate and promote the osteogenic differentiation of stem cells shows that the simultaneous roles of surface patterns and substrate viscoelastic properties modulate stem cell differentiation toward osteogenic phenotypes. To better understand the regulation of cell differentiation by mechanical signals, Witkowska-Zimny et al. [9] investigated the influence of matrix stiffness (E = 1.46 kPa and E = 26.12 kPa) on the differentiated osteogenic cell line of bone marrow stem cells (BM-MSCs) and bone-derived cells (BDCs) using flexible polyacrylamide substrates coated with collagen. They found that substrate stiffness can regulate osteogenic differentiation, which may depend on commitment to multi- or unipotent target cells. The osteogenic differentiation of BM-MSCs was greater on a rigid substrate than on a soft substrate, whereas the osteogenic differentiation of BDCs did not vary with substrate stiffness. These data highlight the role of external mechanical determinants in stem cell differentiation and may be useful in translating the approach to functional tissue engineering. These previous studies have been investigating cell proliferation and differentiation by culturing stem cells on hydrogel substrates with controllable stiffness. However, it would be particularly important to understand the influence of mechanical properties on stem cell behavior in terms of osteogenic growth factor, as cell behavior may differ depending on substrate stiffness.

It is well known that the ideal biomaterial to form a reliable hydrogel must be biocompatible, biodegradable, and non-toxic to promote cell adhesion, growth, and differentiation [10,11]. Alginate is a natural polysaccharide approved by the FDA and widely used as a biomaterial and bio-ink when loaded with cells [12,13]. Alginate is often combined with bioactive molecules or other polymers to enable cell adhesion and proliferation [14]. The combination of sodium alginate and gelatin makes them useful for cell culture [15,16]. Alginate provides a porous structure that allows the diffusion of nutrients and growth factors, while gelatin offers a favorable surface for cell adhesion and differentiation [17]. Studies have demonstrated that hydrogels composed of alginate and gelatin can promote the osteogenic differentiation of mesenchymal stem cells, contributing to bone formation [15,16]. Therefore, Alg-Gel hydrogels are considered good candidates for osteogenic differentiation due to their biocompatibility and surface properties that promote cell adhesion and differentiation, as well as their ability to induce the differentiation of mesenchymal stem cells into osteoblasts [18,19]. Despite advances in Alg-Gel, few studies have investigated the influence of their stiffness on the expression of the osteogenic markers of DPSC cells, such as osteocalcin, alkaline phosphatase, and type I collagen [16,20]. The expression of these markers is used as an indicator of the osteogenic differentiation of DPSC cells [20]. Therefore, studying the expression of these markers as a function of the stiffness of the alginate/gelatin hydrogel is important in developing a hydrogel that can guide the osteogenic differentiation of DPSCs based on its stiffness [21].

**Figure 1:**
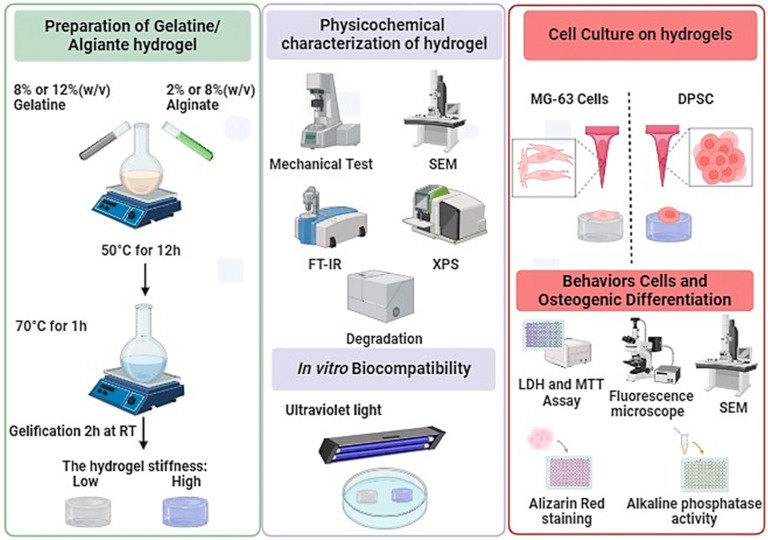
Synthesis, characterization, and cell culture on low- and high-Stiffness hydrogels.

The objective of this study was to differentiate DPSCs and MG-63 (cells derived from osteosarcoma (used as a positive control)) into osteoblast-like cells and to study their interaction with hydrogels of different stiffness (Figure 1). Our approach is based on enhancing the mechanical properties of hydrogels by adjusting the ratios of alginate and gelatin. We have fine-tuned the stiffness of hydrogels to specific targets, notably around 11 ± 1 kPa and 55 ± 3 kPa, enhancing their applicability without affecting cell adhesion, proliferation, or morphology. Interestingly, we observed a significant enhancement in osteogenic differentiation on the stiffer hydrogels, as demonstrated by increased Alizarin Red staining and alkaline phosphatase activity, thus suggesting a potential effect in guiding tissue engineering outcomes. Therefore, this newly developed hydrogel with optimized stiffness can promote the adhesion and growth of osteogenic cells, forming bone tissue for clinical applications.

## 2. Materials and Methods

### 2.1. Materials

Sodium alginate (W201502), gelatin (type A: from porcine skin), calcium chloride dihydrate (ReagentPlus^®^, ≥99.0%), calcium chloride, phosphate-buffered saline (PBS), and ascorbic acid-2-phosphate were obtained from Sigma-Aldrich (St. Louis, MO, USA). For cell culture experiments, Dulbecco’s Modified Eagle Medium F12 (DMEM F12), L-Glutamine (200 mM), Antibiotic/Antimycotic Solution (Pen/Strep/Fungiezone), Fetal Bovine Serum (FBS), and dexamethasone (98%) were purchased from Thermo Fischer Scientific (Mississauga, ON, Canada). Normal human dental pulp stem cells (code 36086) were purchased from Cedarlane Labs (Burlington, ON, Canada). The MG-63 osteoblast cell line (CRL-1427) was obtained from the American Type Culture Collection (ATCC, Manassas, VA, USA).

### 2.2. Preparation of Alginate/Gelatin Hydrogels

Different hydrogels based on varying percentages of Alg-Gel were prepared in this study, namely: 2% (*w*/*v*) alginate and 4% (*w*/*v*) gelatin, 2% (*w*/*v*) alginate and 6% (*w*/*v*) gelatin, 2% (*w*/*v*) alginate and 8% (*w*/*v*) gelatin, 4% (*w*/*v*) alginate and 6% (*w*/*v*) gelatin, 4% (*w*/*v*) alginate and 8% (*w*/*v*) gelatin, 6% (*w*/*v*) alginate and 8% (*w*/*v*) gelatin, 8% (*w*/*v*) alginate and 12% (*w*/*v*) gelatin, and 12% (*w*/*v*) alginate and 20% (*w*/*v*) gelatin. Both hydrogels were prepared in PBS (0.1 M, pH 7.4). The different solutions were placed in a water bath at 50 °C overnight to dissolve the polymers and homogenize the solutions. Then, a pasteurization process was performed at 70 °C for 1 h for all the experiments. To obtain hydrogel samples, 3 mL of Alg-Gel solution was added to a six-well plate (Sarstedt, St-Leonard, QC, Canada) and left at room temperature for 2 h to promote complete gelation. The hydrogel samples were then sliced with a sterile scalpel blade into 1 × 1 cm squares. All the samples were immersed in a 2% CaCl_2_ solution for cross-linking for 1 h. Finally, the samples were washed with deionized water to remove excess CaCl_2_ and were stored at 4 °C for further use.

### 2.3. Hydrogels Physicochemical Characterizations

The surface morphology of the hydrogels was analyzed by scanning electron microscopy (SEM) on a Quanta 250, FEI Company Inc (Hillsboro, OR, USA). (Thermo-Fisher Scientific, Hanover Park, IL, USA), operated at 5 kV/6 spot in secondary electron mode. Before scanning, all the samples were immersed in liquid nitrogen for 60 s, freeze-dried for 24 h, and sputter-coated with gold nanoparticles for 2 min to avoid surface burning.

The complementary analysis of the functional groups on hydrogels was determined by Fourier-transform infrared-attenuated total reflectance (FTIR-ATR) spectroscopy using an Agilent Cary 660 FTIR (Agilent Technologies, Santa Clara, CA, USA). All the measurements were obtained in absorbance mode and 128 scans were recorded between 500 and 4000 cm^−1^ with a spectral resolution of 4 cm^−1^. All the samples were partially dried at room temperature and measured to confirm the chemical composition. The chemical composition of the scaffolds and the cross-linking with CaCl_2_ was assessed by X-ray photoelectron spectroscopy (XPS) using a PHI 5600-ci equipment (Physical Electronics, Chanhassen, MN, USA). Before the measurements, the samples were freeze-dried for 24 h, and each sample was scanned at three different positions to observe the homogeneity of the cross-linking.

### 2.4. Rheological Characterizations

All the rheological measurements were performed using a rheometer (DHR-3, TA Instruments, New Castle, DE, USA) with parallel plate geometry and a diameter of 20 mm. Circular hydrogels were obtained from the cross-linked samples by puncturing the hydrogels with a steel hollow punch with an internal diameter of 20 mm. Sandpaper (80 grit) was pasted on the surface of the parallel plates to avoid the samples’ slipperiness during the measurements [22]. Storage modulus (G′) and loss modulus (G″) were measured on an amplitude sweep oscillatory test ranging from 0.1 to 10% strain using an angular frequency of 1 rad/s at 37 °C. A thermal sweep test in the linear viscoelastic range at 0.5% strain with an angular frequency of 1 rad/s was performed from 10 °C to 40 °C. Moreover, the cross-linking behavior was tested through frequency sweep experiments from 0 to 100 rad/s applying 0.5% strain at 37 °C.

### 2.5. Swelling Properties

To determine the swelling of the hydrogel, we measured its initial dry weight (W_0_) in milligrams (mg). The hydrogels were then lyophilized to eliminate all the water. The lyophilized hydrogels were then immersed in 3 mL of PBS (pH 7.4) for 24 h at 37 °C to reach swelling equilibrium. After this period, each swollen hydrogel was weighed (W_1_) after removing the excess solution using filter paper. Finally, the swelling ratio of the hydrogels was calculated using the following formula: swelling rate (%) = (W_1_ − W_0_)/W_0_ × 100%.

### 2.6. Degradation Behavior

The hydrogel degradation was investigated by immersing the samples in 3 mL of PBS and incubating them at 37 °C. The initial mass (W_0_) was measured, and the hydrogels were weighed at different times (W1: 7, W2: 14, and W3: 21 days). The remaining mass (wet weight, %) was calculated using the following equation: remaining (%) = W_1_/W_0_ × 100%.

### 2.7. Cell Culture

To avoid cell culture contamination, the hydrogels were soaked in 70% ethanol in the presence of ultraviolet (UV) light for 30 min and then washed with sterile PBS [23,24]. The sterile Alg-Gel hydrogels were placed in the wells of six-well plates and incubated in DMEM/F12 supplemented with 1% (*v*/*v*) penicillin/streptomycin at 37 °C overnight (hydrogel preconditioning) before cell seeding. For each hydrogel, 50 µL of culture medium containing 2 × 10^5^ cells was applied onto the hydrogel surface and placed in a six-well culture plate. Subsequently, the cell-seeded hydrogels were placed in a humidified incubator set at 37 °C and 5% CO_2_ for 2 h for optimal cell adhesion.

Following this incubation, 3 mL of culture medium (DMEM/F12 supplemented with 10% FBS and 1% (*v*/*v*) penicillin/streptomycin) was added to each cell-seeded hydrogel to provide an appropriate cellular growth environment. The cells were then cultured on the hydrogels for 7, 14, and 21 days, allowing for the study of cellular behavior over time.

To induce the differentiation of DPSC cells into osteoblasts, the culture medium was supplemented with 100 μM l-ascorbic acid 2-phosphate and 10^−7^ M dexamethasone, forming an osteoinductive medium (OM). This stimulation was essential for promoting cell development in the desired direction.

The DPSCs used were from passages 4 to 6, while the MG-63 cells were from passages 10 to 12. This selection of cell passages ensured the reliable characterization of cell attachment, proliferation, and differentiation, thereby ensuring the quality of the results obtained in our study.

#### 2.7.1. Osteoblast Adhesion

The hydrogel samples were carefully placed into the wells of a 12-well plate. Subsequently, the MG-63 and DPSCs cells were seeded at a density of 5 × 10^4^ cells per well. These cell cultures were then incubated for specific durations of 1, 3, and 7 days to facilitate cell adhesion to the hydrogels and growth. At the end of each culture period, the hydrogels were gently washed twice with PBS to remove any residual culture medium or non-adherent cells.

The cells were then fixed with a 4% formaldehyde solution (Sigma Aldrich, Burlington, MA, USA) at room temperature for 60 min. This fixation step stabilized the cellular structure in preparation for subsequent procedures. Once fixed, the cells were washed with PBS to remove any excess fixative.

The fixed cells were stained with Hoechst 33342 dye (Thermo Scientific, Norristown, PA, USA) for 15 min to visualize the cellular nuclei. This nucleus-specific dye allows for the precise observation of the cells under the microscope. Following staining, the hydrogels were washed three times for two minutes each with distilled water to remove any excess dye.

Finally, cell images were acquired using an Olympus FSX100 fluorescence microscope. The images were captured at high resolution and analyzed using the FSX-BSW imaging software (FSX100 software, FSX-BSW(03.02.12)) (Olympus, Tokyo, Japan) to evaluate the cellular distribution and density of the hydrogels.

#### 2.7.2. Cell Viability

Following a culture period of 1, 3, 7, 14, and 21 days of the DPSCs and MG-63 cells on the Alg-Gel hydrogels, we assessed cell viability using two methods: cell counting and the MTT (3-(4,5-dimethylthiazol-2-yl)-2,5-diphenyl-2H-tetrazolium bromide) assay. Cultures for each technique were prepared separately to ensure accurate evaluation. Initially, 5 × 10^4^ cells were seeded onto each hydrogel in wells of a 12-well plate, followed by incubation for the specified durations. At the end of each culture period, hydrogels were washed twice with PBS to remove any residual culture medium or non-adherent cells.

For cell quantification on the hydrogels, cells were detached using a 0.25% (*w*/*v*) trypsin-EDTA solution and washed twice with the culture medium. Subsequently, the cells were suspended in 1 mL of the culture medium and thoroughly mixed. To determine cell counts, 10 μL of each cell suspension was mixed with 10 μL of trypan blue solution and transferred to a hemocytometer chamber for counting under an optical microscope to distinguish viable cells (unstained by trypan blue) from dead cells (trypan blue-stained cells).

Cell viability was further evaluated using the MTT assay following the manufacturer’s protocol (Roche Diagnostics, Mannheim, Germany). This assay measures the ability of viable cells’ mitochondrial dehydrogenase to reduce MTT to purple formazan crystals. After each culture period, the medium was supplemented with 10% MTT solution and incubated at 37 °C for 3 h. Two washes were allowed to remove excess MTT, and the formazan present in live cells was solubilized in an isopropanol/0.04% HCl solution. Finally, triplicate 200 μL aliquots of the supernatant were transferred from each hydrogel to wells of a 96-well flat-bottom plate, and the absorbance at 550 nm of the formazan dye was determined using a microplate spectrophotometer reader (X-Mark microplate spectrophotometer, Bio-Rad, Hercules, CA, USA).

#### 2.7.3. Cytotoxicity Assessment

Cytotoxicity was examined using a lactate dehydrogenase (LDH) assay. The DPSCs and MG-63 cells were cultured on hydrogels in 12-well plates (5 × 10^4^ cells/well) for 1, 3, 7, 14, and 21 days [25]. After each culture period, the medium was collected and stored at −80 °C to assess cellular damage, which was measured by LDH release. Briefly, 10 µL of the culture supernatant was transferred to a 96-well plate, and the enzymatic reaction was performed following the instructions of the LDH cytotoxicity assay kit (Roche Diagnostics, Manheim, Germany). Next, 100 µL of the LDH reaction mixture was added to each well, and the plate was incubated in the dark for 30 min at room temperature. After that, 10 µL of a stop solution was added to stop the reaction. The absorbance was measured at 450 nm using an X-Mark microplate spectrophotometer (Bio-Rad). The experiment included negative, positive, and background controls for total LDH activity.

#### 2.7.4. Cell Morphology

SEM was used to study the morphology of the DPSCs and MG-63 cells after 7 days of culture on the hydrogels. Following the cell culture, the hydrogels were fixed in a solution of 0.2% alcian blue, 0.2% red ruthenium, and 2.5% glutaraldehyde in 0.1 M cacodylate buffer (pH 7.2) for 4 h at room temperature. This step was repeated one more time. After fixation, the samples were sputtered with gold and observed under a JEOL 6360 LV scanning electron microscope (Soquelec Inc., Montreal, QC, Canada) at an accelerating voltage of 15 kV.

### 2.8. Osteogenic Activity

The DPSCs and MG63 cells (at a density of 2 × 10^5^ cells) were cultured on the different hydrogels for 7, 14, and 21 days. At the end of each culture period, the supernatant was collected and used to measure the activity of alkaline phosphatase (ALP) (Alkaline Phosphatase Assay Kit, No. ab83369, Abcam, Waltham, MA, USA) according to the manufacturer’s protocol. Briefly, 80 µL of each supernatant was transferred, in technical triplicates, to the wells of a 96-well flat-bottom plate and was incubated with 50 µL of 5 mM pNPP solution for 60 min at room temperature, followed by adding 20 µL of stop solution to stop the conversion of p-nitrophenol into p-nitrophenylate. The absorbance was determined by a BioRad microplate reader at 405 nm. The Alg-Gel hydrogels were fixed with 4% formaldehyde for 60 min at room temperature and then stained with Alizarin Red S Staining (ARS) (Sigma-Aldrich, St. Louis, MO, USA) to evaluate the mineralization after 7, 14, and 21 days. Briefly, 500 µL of cetylpyridinium chloride was added to each well and incubated for 15 min at room temperature to dissolve the calcium nodules. The OD value was measured at a wavelength of 550 nm using a BioRad microplate reader. The results were normalized to a determined number of viable cells (10^6^ cells).

### 2.9. Statistical Analysis

The values were reported as the means ± SDs for each analysis based on six experiments. To test the significance of the observed difference between the experimented groups, an unpaired Student’s *t*-test (except otherwise stated) was applied, with a value of *p* < 0.05 considered to be statistically significant.

## 3. Results and Discussion

### 3.1. Preparation and Characterization of Alg-Gel Hydrogels

**Figure 2:**
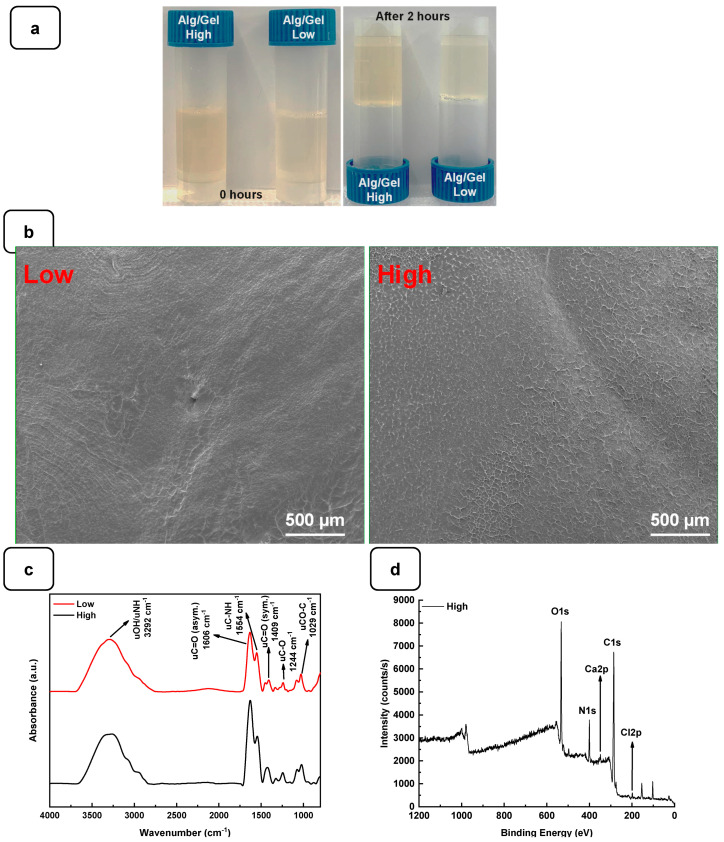
(**a**) Gelification of Alg-Gel hydrogels after 2 h at room temperature. (**b**) SEM images of low and high Alg-Gel hydrogels. (**c**) FTIR-ATR spectra of low and high Alg-Gel hydrogels (**d**) XPS analysis of high hydrogel with CaCl_2_.

To examine the gelation of the hydrogels, the photographs of the vials in the reverse reaction mode were taken before and after the completion of the process, as shown in Figure 2a. No movement was observed in the solution after 2 h of incubation at 22 °C, indicating complete gelation. The structural analysis using SEM showed that both hydrogels exhibited a flat and homogeneous surface with no visible pores (Figure 2b).

The interaction between the alginate and gelatin contributing to the hydrogel formation was monitored by FTIR-ATR spectroscopy (Figure 2c), confirming the presence of specific absorption bands assigned to the vibrations of the corresponding alginate and gelatin functional groups, as reported previously [24]. The absorption peaks at 1606 cm^−1^ and 1406 cm^−1^ in the infrared spectrum of pure alginate were attributed to the asymmetric stretching vibrations of the alginate groups. In addition, the absorption band at about 1400 cm^−1^ represents the carboxyl group of gelatins. The two absorption peaks at 1635 cm^−1^ and 1534 cm^−1^ correspond to gelatin’s amide I and amide II bands. These changes indicate a strong molecular interaction between the alginate and gelatin chains through the self-organization of polyelectrolyte complexes, including hydrogen bonding and electrostatic attraction [17,26]. The surface XPS survey spectrum of the hydrogels is shown in Figure 2d and Figure S1. C and N are components of Alg and Gel, while Ca refers to calcium chloride and is used as a cross-linking agent [24].

**Figure 3:**
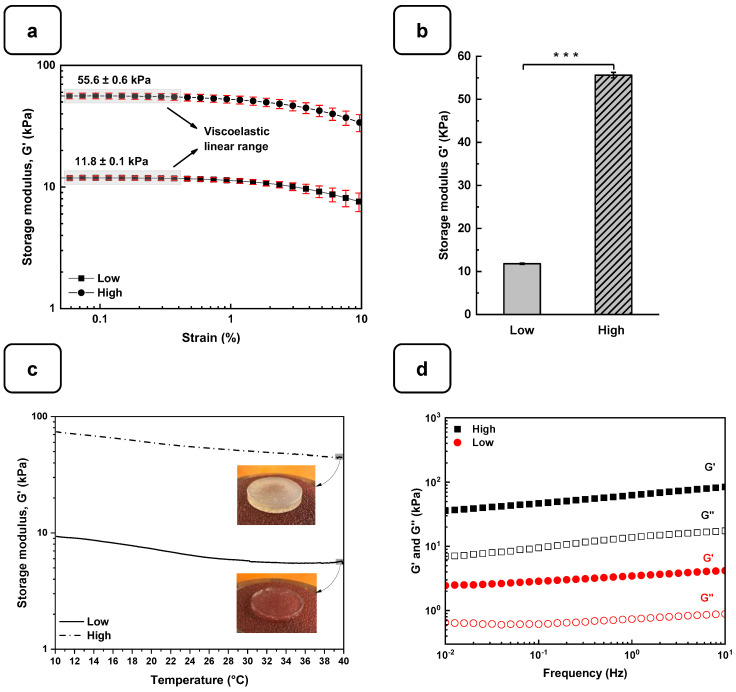
Rheological characterization of the Alg-Gel hydrogels for the low- and high-stiffness formulations. (**a**) The evaluation of the G′ and G″ by strain amplitude sweep at 37 °C. (**b**) Average elastic modulus measured from the linear viscoelastic range. (**c**) The evaluation of the hydrogel’s stability by thermal sweep tests. (**d**) The frequency sweep tests of low- and high-stiffness hydrogels at 37 °C. *** *p* < 0.001.

The rheological studies for the Alg-Gel hydrogels were performed to assess the viscoelastic behavior of the cross-linked hydrogels and obtain two types of hydrogels with low and high stiffness properties. In this case, low-stiffness hydrogels refer to Alg-Gel ratios of 2%/8%, while the ratios for high-stiffness hydrogels correspond to 8%/12%. These two hydrogels were selected from various hydrogels prepared in this study with different percentages of alginate and gelatin (Figure S2). Strain sweep tests ranging from 0.1 to 10% show the linear viscoelastic region of both formulations at low shear stress and 37 °C (Figure 3a). The storage modulus (G′) obtained from the viscoelastic region (Figure 3b) shows a significant difference for the high-stiffness hydrogels at around 55.6 ± 0.6 kPa compared to 11.8 ± 0.1 kPa for the low-stiffness hydrogels. Similar stiffness values were reported by Chen et al. [27] using 20% (*w*/*v*) of methacrylated gelatin (GelMA) and 3% (*w*/*v*) of sodium alginate. In that study, the authors promoted a stiffness increase in the dynamic hydrogel from 10 to 68 kPa using UV, CaCO_3_, and CaCl_2_ as cross-linkers. Our study showed good reproducibility of the formulations tested to obtain two types of hydrogels with high and low stiffness using as low as 2% (*w*/*v*) of CaCl_2_ for 1 h to cross-link the alginate.

On the other hand, the thermal sweep experiments of the scaffolds confirmed that the time and cross-linking concentration were enough for both formulations to obtain a hydrogel that does not have a significant change in stiffness, as is shown in Figure 3c. The volume of the CaCl_2_ solution and the time used to cross-link all the hydrogels were sufficient to promote an optimal Ca^2+^ ion penetration into the hydrogel structure, as reported previously [28]. Additionally, a frequency sweep test was performed to evaluate the impact of the cross-linking conditions on the storage and loss modulus. Figure 3d shows no crossover point at higher frequency ranges. It suggests that the G′ and G″ values are independent of the frequency, a typical behavior of chemically cross-linked hydrogels [29]. Taking into consideration the temperature sensitivity and the concentration of the gelatin matrix in the scaffolds, the lack of a crossover point could be due to the good distribution and entanglement of the cross-linked polymeric chains of Alg with the Gel chains, avoiding their free movement to flow even at 37 °C. This behavior could be compared with the results obtained by Gregory et al. [30]. In their study, the frequency sweep tests presented an increase in G′ and G″ proportional to the frequency with no crossover point when the Alg is at a high molecular weight.

Another important factor affecting the mechanical properties of the gels is the tendency to swell. Figure S3a shows that the average swelling rates of the low and high hydrogels were 20 ± 3% for “high” and 35 ± 2% for “low”. The stability of the hydrogels in PBS at 37 °C is shown in Figure S3b. After 7 days of incubation in PBS at 37 °C, there was no significant stiffness difference between the two hydrogels. However, after 14 days, a difference was observed, with a higher degradation rate for the low-stiffness hydrogel. This observation was confirmed after 21 days of incubation, with respective degradation percentages of 53 ± 5% and 82 ± 2% for the two hydrogels. These results demonstrate that the degradation rate and swelling rate depend on the amount of alginate and gelatin and the absence of pores, which can increase the degradation and swelling rates [31].

### 3.2. Biological Performances of Alg-Gel Hydrogels

**Figure 4:**
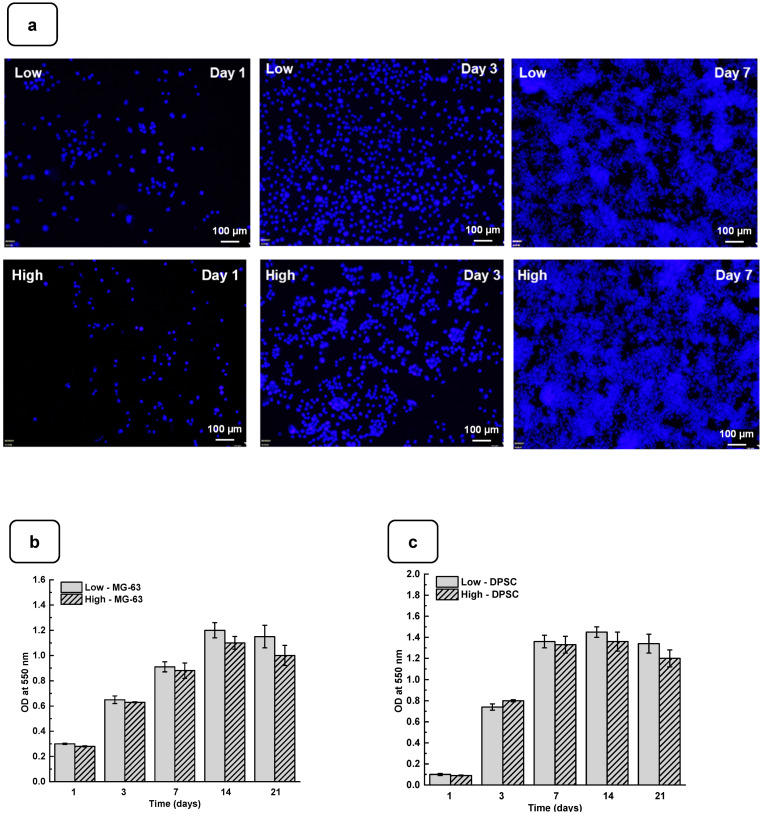

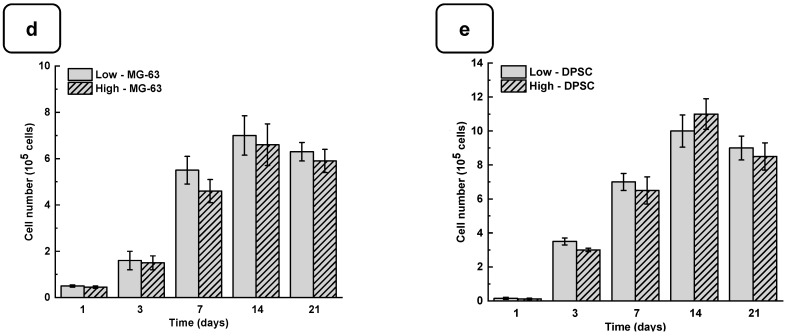
(**a**) DPSC cells cultured for 1, 3, and 7 days on low and high hydrogels and stained with Hoechst dye. MTT assay for cells cultured for 1, 3, 7, 14, and 21 days on low and high hydrogel: (**b**) MG-63 cells and (**c**) DPSCs. Cell counts of (**d**) MG-63 and (**e**) DPSCs were cultured for 1, 3, 7, 14, and 21 days on hydrogel. Data points are means of 3 independent experiments in duplicate (±SD).

Adhesion is one of the major factors revealing cell behavior. If adherent cells cannot attach due to inappropriate conditions, they will not grow, and finally, they will die. Thus, the evaluation of osteoblast adhesion to the Alg-Gel hydrogels is important to demonstrate whether the hydrogels are suitable for cell culture. As shown in Figure 4a and Figure S4, both the DPSCs and MG63 cells had good adhesion to all the tested hydrogels. The Hoechst staining showed stained cells all over the surface of each hydrogel. The stained cell density was increased from day one to day 21 of culture, suggesting cell adhesion and proliferation. To confirm such observation, we performed an MTT assay to measure cell growth viability at different culture periods (Figures S5 and S6). As reported in Figure 4b,c the hydrogels promoted osteoblast proliferation. Indeed, with DPSCs, absorbances after 7 days of culture were 1.33 ± 0.04 with the “low” hydrogels and 1.29 ± 0.06 with the “high” hydrogels. Similar results were obtained with the MG63 cells cultured for 7 days on the hydrogels, with absorbances ranging from 0.95 ± 0.02 with the “low” hydrogels and 0.82 ± 0.01 with the high hydrogels. At 21 days post-culture, DPSCs showed increased absorbances with 1.32 ± 0.09 for the “low” hydrogels and 1.11 ± 0.07 for the “high” hydrogels. MG63 also showed better growth at 21 days with an absorbance of 1.12 ± 0.08 with the low-stiffness hydrogels and 1.05 ± 0.07 with the high-stiffness hydrogels. Overall, this study demonstrated that hydrogel stiffness did not affect osteoblast cell adhesion and proliferation (MG-63 and DPSCs). The proliferation of osteoblasts on the alg-gel hydrogels was confirmed by trypan blue exclusion assay. Figure 4d,e show that after one day of culture on the hydrogels, the cell count of DPSCs on the low and high hydrogels is 1.5 ± 0.3 × 10^4^ and 1.1 ± 0.2 × 10^4^, respectively. With MG-63, the live cell numbers were 5.2 ± 0.1 × 10^4^ on the low and 4.7 ± 0.4 × 10^4^ on the high hydrogels. After 7 days of culture, the live cell numbers significantly increased, reaching 3.4 ± 0.2 × 10^5^ and 3.0 ± 0.1 × 10^5^ DPSCs, while with the MG-63 we had 6.5 ± 0.2 × 10^5^ and 6.1 ± 0.2 × 10^5^ for the low and high hydrogels, respectively. At 21 days of culture, there was no increase in the cell number compared to 7 and 14 days, suggesting cell growth saturating due to the absence of free space for them to proliferate. Our results with Hechest sating, MTT assay, and Trypan blue exclusion assay confirmed that these hydrogels promote cell adhesion, viability, and proliferation with no toxic effect. Indeed, the toxicity was evaluated by an LDH release assay and showed (Figure S7) no significant increase in the LDH activity when comparing the cell culture or not on each hydrogel type.

As the hydrogels were not toxic to the osteoblasts, we also compared the cell adhesion and proliferation obtained with low and high hydrogel stiffness, showing no significant differences. The absence of a significant difference in cell adhesion and proliferation on hydrogels despite a significant difference in stiffness between the low and high hydrogels can be explained by the fact that other properties of the hydrogels besides stiffness can influence cell adhesion and proliferation. Indeed, the hydrogels’ chemical composition, texture, and permeability can all play a role in the ability of cells to attach and grow on the surface of the hydrogels [32]. As our hydrogels contained gelatin derived from collagen, a significant component of the extracellular matrix of tissues, the gelatin can provide a favorable substrate surface for cell adhesion and promote cell growth [33]. Moreover, it is possible that the difference in stiffness between the hydrogels is not significant enough to have an impact on cell adhesion and proliferation [34]. Finally, it is also possible that the DPSCs and MG-63 used in the experiment have different tolerances to hydrogel stiffness. Some cells may be more sensitive than others to changes in stiffness, which could explain why we did not observe a significant difference in cell adhesion and proliferation on low and high hydrogel stiffness, as reported previously [35].

### 3.3. Cell Morphology

**Figure 5:**
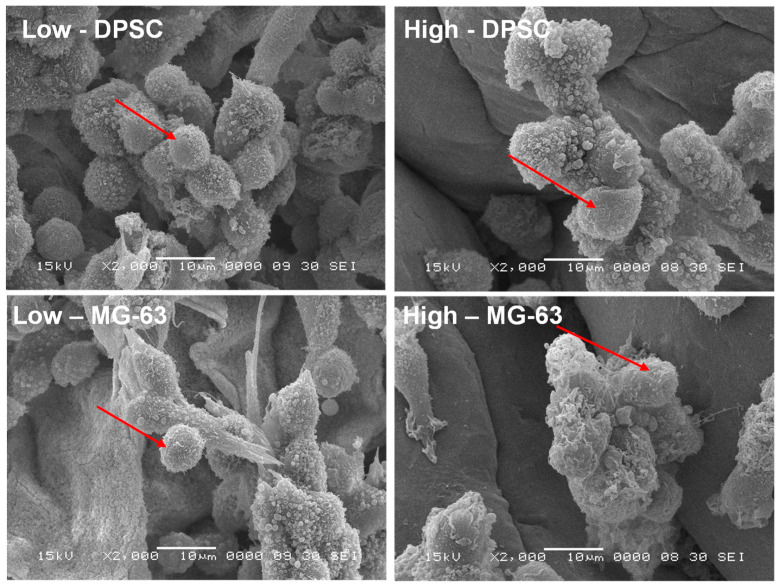
SEM images of DPSCs and MG-63 cells adhering to two hydrogels of different stiffness after 7 days of incubation, as observed by SEM. Arrows indicate the cells adhering to the hydrogels.

The adhesion to and growth on an extracellular matrix may modulate the cell morphology. For this purpose, we analyzed the osteoblast ultrastructure after growth on the Alg-Gel hydrogels and subjected them to SEM analysis. As shown in Figure 5, the cells exhibited rounded and spherical morphology and were growing as grapes as bone-like nodules. These results suggest that the stiffness of hydrogels do not negatively influence the cell shape and support those showing that cells cultured on soft hydrogels exhibit a rounded and spherical shape [34]. Overall, our study showed the possibility of optimizing Alg-Gel hydrogel stiffness without affecting the cell morphology of DPSCs or MG-63.

However, it is possible that the stiffness of the hydrogels does not directly influence the morphology of these cells because the cells have an intrinsic ability to adapt their shape and structure to their environment [36,37]. Indeed, cells can sense their environment through surface receptors, such as integrins, which allow them to detect the mechanical properties of their environment, including the stiffness of hydrogels [38]. The cells can then adapt their adhesion and motility in response to these mechanical properties [39,40]. In addition, it is important to note that hydrogel stiffness may still indirectly affect the cells by altering the cell’s response to biochemical signals and influencing cell differentiation [41].

### 3.4. Mineralization of Cells on Hydrogels

**Figure 6:**
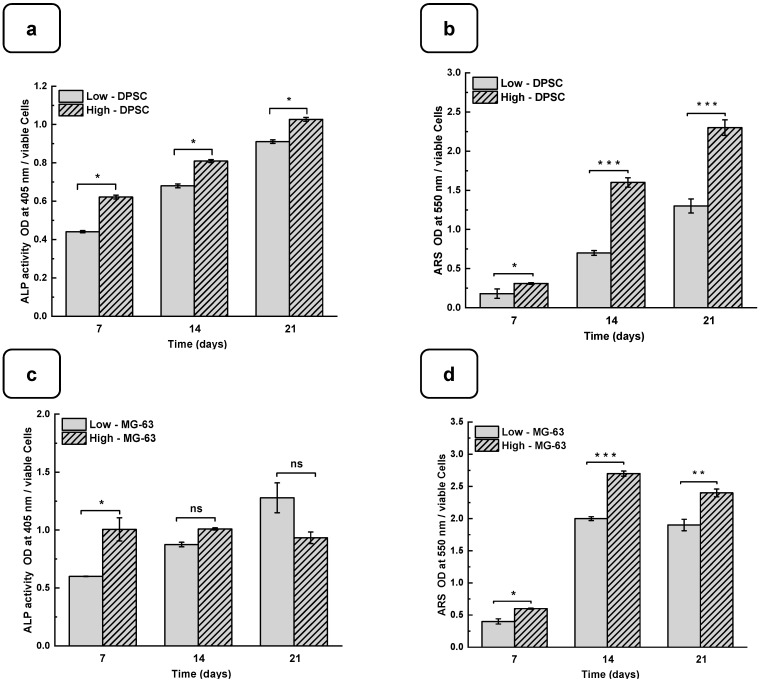

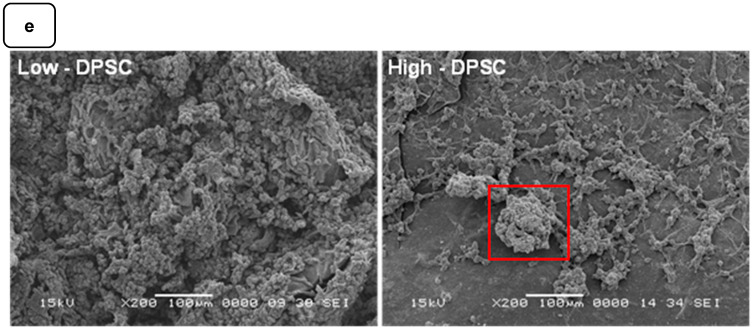
DPSC differentiation into osteoblast-like cells. (**a**) Alkaline phosphatase activity. (**b**) ARS staining and CPC extraction. MG-63 differentiation into osteoblast-like cells. (**c**) Alkaline phosphatase activity. (**d**) ARS staining and CPC extraction. (ODs were normalized by 10^6^ viable cells). (**e**) SEM photomicrographs of the osteoblast-type cells cultured for 7 days on low and high hydrogels. Nodular accretions with a diameter close to 100 µm were observed on the cells on the seventh day of culture with the high hydrogel. ns: non significatif, * *p* < 0.05, ** *p* < 0.01, *** *p* < 0.001. The red square shows the formation of nodules.

DPSCs and MG-63 mineralize in vitro and produce large extracellular calcium deposits. ALP activity and cell mineralization were used to assess osteoblast differentiation with low and high hydrogels. Figure 6a,b show the ALP activity of DPSCs and MG-63 cells cultured on low and high hydrogels. All the results are normalized to 10^6^ viable cells. ALP activity on both the hydrogels increased with the duration of the culture period. Furthermore, ALP activity was higher on high hydrogels than on low hydrogels. A significantly high ALP activity was obtained with the MG63 being cultured for 7 days on the high compared to the low hydrogels. However, ALP activity did not differ at 14 and 21 days of culture. This can be explained by the fact that ALP is an early osteogenic marker and that the MG-63 cells are a positive control in our study [42,43]. Interestingly, the use of DPSCs showed a significant increase in ALP activity at all the tested culture periods, suggesting the high osteogenic capacity of the Alg-Gel hydrogels we designed, thus enabling bone formation.

Such bone formation can be evaluated using calcium deposition through ARS staining [44,45]. Figure 6c,d show that calcium deposition on low and high hydrogels increased significantly from day 7 to day 21. Indeed, the CPC absorbance increased from 0.21 ± 0.01 on day 7 to 1.25 ± 0.05 on day 21 when the DPSCs were cultured on the low hydrogels. As for the high hydrogels, the CPC absorbance increased from 0.35 ± 0.02 on day 7 to 2.45 ± 0.04 on day 21. Similarly, for MG-63, CPC absorbance changed from 0.41 ± 0.01 and 0.65 ± 0.02 on day 7 to 1.92 ± 0.04 and 2.40 ± 0.02 on day 21 and on the low and high hydrogels, respectively. The CPC activities are about twice as high in high than in the low hydrogels for both cell types, confirming that the high-stiffness hydrogels promote osteogenic differentiation [37,46]. To confirm the osteogenic activity of the Alg-gel hydrogels, we performed an SEM analysis, which showed bone nodule formation (Figure 6e) [44].

Indeed, in the SEM photomicrographs, there were specific structures known as mineral nodules on the hydrogels after a 7-day culture period of the osteoblasts. These nodules, appearing in specific areas indicated by the squares on the images, exhibit an appearance reminiscent of cauliflower and have an average diameter of about 100 µm. Higher bone nodules were obtained compared to the low-stiffness hydrogels (Figure 6e). Such mineralization rate was obtained at the different tested times, showing that the high hydrogels exhibited a significantly higher mineralization rate than the low hydrogels. Such observation highlights the fact that the stiffness of the hydrogel plays a crucial role in the mineralization process, suggesting that the harder hydrogels provide a more osteoconductive environment for bone nodule formation. Consequently, this finding underscores the importance of choosing the correct hydrogel stiffness, especially in biomedical applications where mineralization is crucial, such as bone regeneration.

Cell adhesion is a critical initial process when stem cells come into contact with a hydrogel [47]. Studies have shown that the stiffness of the hydrogel influences the cells’ ability to attach to the matrix [47]. Generally, stem cells tend to adhere more effectively to hydrogels with a stiffness similar to their natural microenvironment [48]. Stronger cell adhesion promotes interaction between cells and the extracellular matrix, which can influence subsequent cell differentiation [48]. Cell morphology is also closely linked to hydrogel stiffness. When cultured on rigid hydrogels, stem cells spread and form more developed cytoskeletal structures, mimicking their natural morphology in tissues [49].

In contrast, on softer hydrogels, cells tend to adopt a more rounded and less spread-out shape [49]. This variation in cell morphology can influence cellular signaling, cell polarity, and stem cell differentiation [49]. Hydrogel stiffness can also affect the proliferation of stem cells [7]. Studies have shown that stem cells proliferate more on stiffer hydrogels than on the softer ones [7]. This may be attributed to the better transmission of mechanical signals from the rigid substrate to the cells, promoting cellular proliferation [7]. It is worth noting that cellular responses to hydrogels of varying stiffness may vary depending on the type of stem cell, the stage of cell differentiation, and other signals present in the microenvironment [7]. Therefore, it is important to carefully characterize the effects of hydrogel stiffness on specific stem cells and to adapt the properties of the hydrogel accordingly to achieve the desired outcomes regarding cell differentiation and proliferation [50].

The effect of hydrogel stiffness on the osteogenic differentiation of cells has been extensively studied in the scientific literature [34]. Several studies have demonstrated that hydrogel stiffness can directly influence cell differentiation into bone cells [34]. For example, Engler et al. [51] showed that MSCs placed on polyacrylamide hydrogel substrates with varying stiffness exhibited enhanced osteogenic differentiation on stiffer hydrogels. They observed a higher expression of specific osteogenesis markers, such as osteocalcin and osteopontin, on stiffer hydrogels. Similarly, other studies have confirmed the effect of hydrogel stiffness on osteogenic differentiation. Trappmann et al. [40] demonstrated that human embryonic stem cells (hESCs) cultured on stiffer hydrogels promoted osteoblast differentiation, while softer hydrogels favored chondrocyte differentiation. It is noteworthy that hydrogel stiffness can mimic the mechanical properties of bone cells’ natural in vivo environment. For instance, bone tissue is relatively rigid, while fatty tissue is softer. Therefore, by mimicking the stiffness of bone tissue, hydrogels can provide a physiologically relevant environment for osteogenic differentiation. In conclusion, hydrogel stiffness plays an essential role in the osteogenic differentiation of cells. However, it is important to note that other factors, such as the chemical composition of hydrogels and the presence of biochemical signals in the environment, can also interact with stiffness to influence cell differentiation [52].

Our study results on the effect of the Alg-Gel hydrogel stiffness on the osteogenic differentiation of DPSCs offer promising insights into bone tissue engineering. Firstly, it is noteworthy that the study addressed the optimization of hydrogel stiffness to guide osteogenesis. By selecting two hydrogels with different stiffness levels, the study could precisely examine the impact of stiffness on critical parameters such as cell attachment, morphology, proliferation, and the osteogenic differentiation of DPSCs. The results demonstrated that hydrogels with higher stiffness promoted enhanced osteogenic activity compared to those with lower stiffness. This suggests a crucial role of hydrogel stiffness in modulating cellular differentiation towards the osteoblastic phenotype.

Furthermore, the in-depth analyses of the physical and chemical properties of the hydrogels provided valuable insights into their behavior under cell culture conditions. Another important observation is that despite the difference in stiffness, both hydrogels exhibited good cell adhesion and proliferation of DPSCs. This indicates that the modulation of hydrogel stiffness can be achieved without compromising other essential cellular behaviors, which is crucial for their potential application in tissue engineering. Lastly, this study underscores the importance of the precise characterization and optimization of Alg-Gel hydrogel stiffness to guide in vitro osteogenesis effectively. These findings could significantly affect the development of novel therapeutic strategies for bone regeneration and tissue repair.

## 4. Conclusions

This study highlights the significant impact of the mechanical properties of hydrogels, particularly their stiffness, on the fate of DPSCs. While variations in hydrogel stiffness did not significantly affect DPSC adhesion, proliferation, or morphology, they influenced their osteogenic potential. Hydrogels with greater stiffness demonstrated an ability to enhance the osteogenic activity of DPSCs, suggesting a promising avenue for guiding in vitro osteogenesis without the need for chemical-inducing agents. This advancement opens new perspectives for applying mechanobiology in regenerative medicine, particularly in developing more natural and less invasive bone regeneration strategies. The platforms based on the optimized Alg-Gel hydrogel could thus play a crucial role in future studies in cellular mechanobiology and clinical applications in tissue engineering and regenerative medicine.

## Data Availability

Data contained within the article.

## Supplementary Materials

The following supporting information can be downloaded at: https://www.mdpi.com/article/10.3390/biomedicines12071510/s1, Figure S1. XPS analysis of Low Alg-Gel scaffolds with CaCl_2_. Figure S2. Optimization of stiffness as a function of hydrogel composition. Figure S3. Equilibrium swelling studies (a) and Degradation test (b) of Low and High Alg-Gel hydrogels. * *p* < 0.05, ** *p* < 0.01. Figure S4. MG-63 cells cultured for 1, 3 and 7 days on Low and High Alg/Gel scaffolds and stained with Hoechst dye. Figure S5. The growth curves of MG-63 cells on scaffolds (a). The control group and the test group were monitored by MTT assay (b). Each point represents the average absorbance readings of three scaffolds. Figure S6. The growth curves of DPSC cells that were grown on scaffolds (a). The control group and the test group were monitored by MTT assay (b). Each point represents the average absorbance readings of three scaffolds. Figure S7. Cytotoxicity assay of LDH release from DPSCs (a) and MG-63 cells (b) grown on hydrogels.
