# Supplementary material for: Design of Alginate/Gelatin Hydrogels for Biomedical Applications: Fine-Tuning Osteogenesis in Dental Pulp Stem Cells While Preserving Other Cell Behaviors
Source: Biomedicines. 2024 Jul 8;12(7):1510. doi: 10.3390/biomedicines12071510 (PMC11274465; doi:10.3390/biomedicines12071510)
Supplement: Supplementary file 1 [file biomedicines-12-01510-s001.zip › biomedicines-3064106-supplementary.pdf]

SUPPORTING INFORMATIONS

Design of Alginate/Gelatin Hydrogels for Biomedical Applications: Fine-Tuning Osteogenesis in Dental Pulp Stem Cells While Preserving Other Cell Behaviors

Zied Ferjaoui [1,*], Roberto López-Muñoz [2], Soheil Akbari [3], Fatiha Chandad [1], Diego Mantovani [2], Mahmoud Rouabhia [1] and Roberto D. Fanganiello [1]

[1] Oral Ecology Research Group (GREB), Faculté de Médecine Dentaire, Université Laval, Québec City, QC, G1V 0A6, Canada
[2] Laboratory for Biomaterials and Bioengineering, (CRC-Tier I), Department of Min-Met-Materials Eng and Regenerative Medicine, CHU de Quebec, Laval University, Quebec City, QC G1V 0A6, Canada
[3] Département de Génie Chimique, Université Laval, Québec City, QC G1V 0A6, Canada
* Correspondence: zied.ferjaoui.1@ulaval.ca

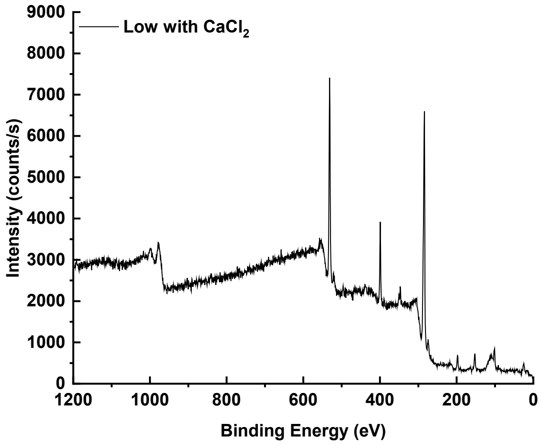

Figure S1. XPS analysis of Low Alg-Gel scaffolds with $CaCl_2$.

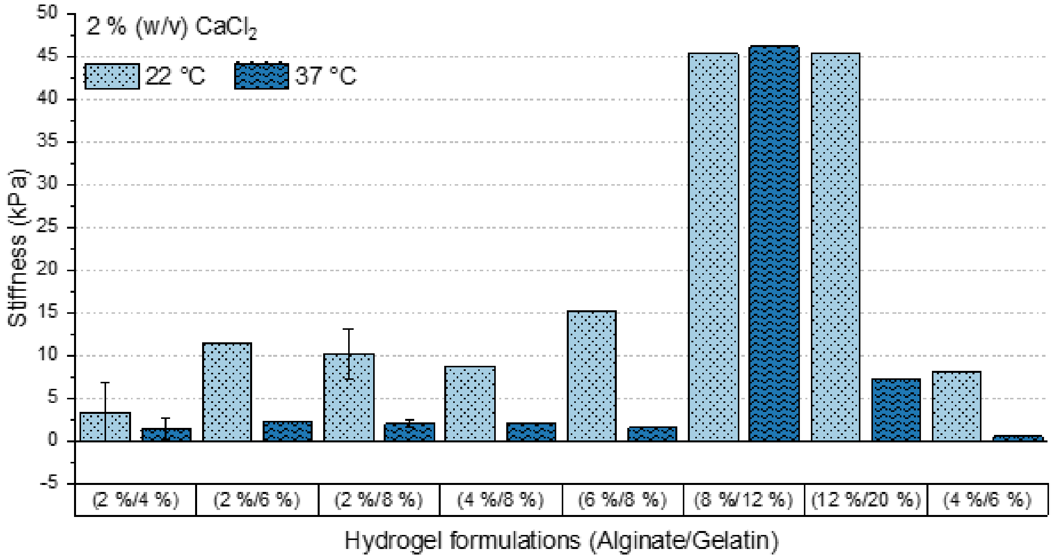

Figure S2. Optimization of stiffness as a function of hydrogel composition.

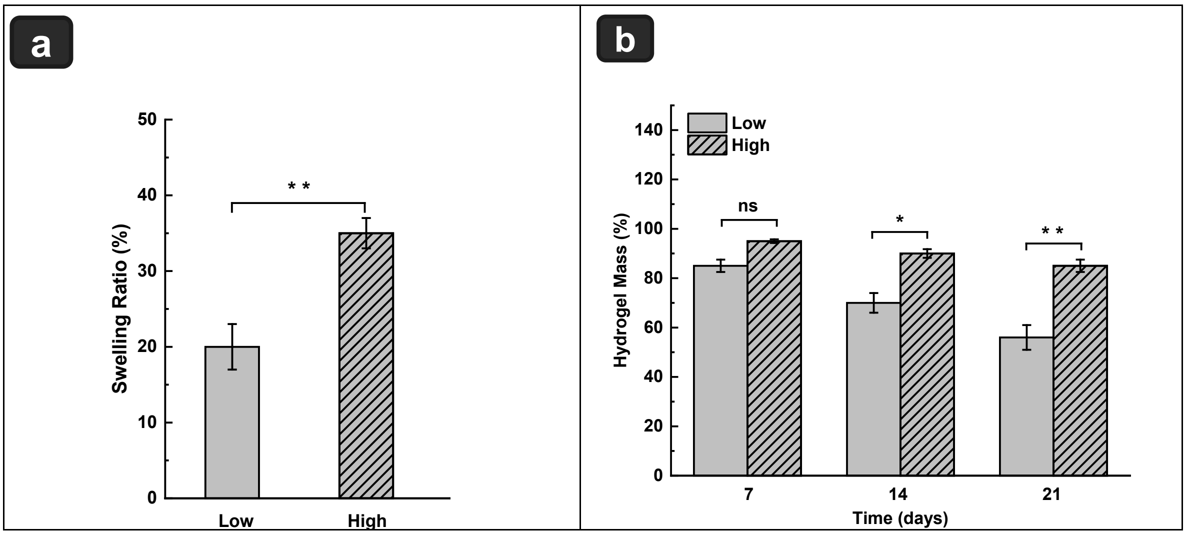

Figure S3. Equilibrium swelling studies (a) and Degradation test (b) of Low and High Alg-Gel hydrogels. *$p < 0.05$, **$p < 0.01$.

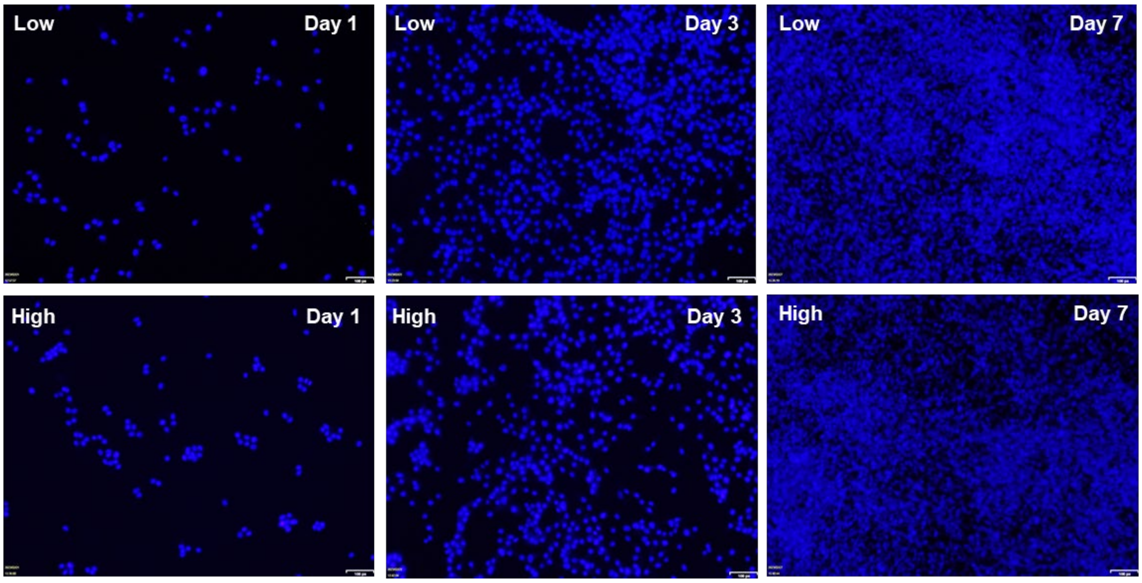

Figure S4. MG-63 cells cultured for 1, 3 and 7 days on Low and High Alg/Gel scaffolds and stained with Hoechst dye.

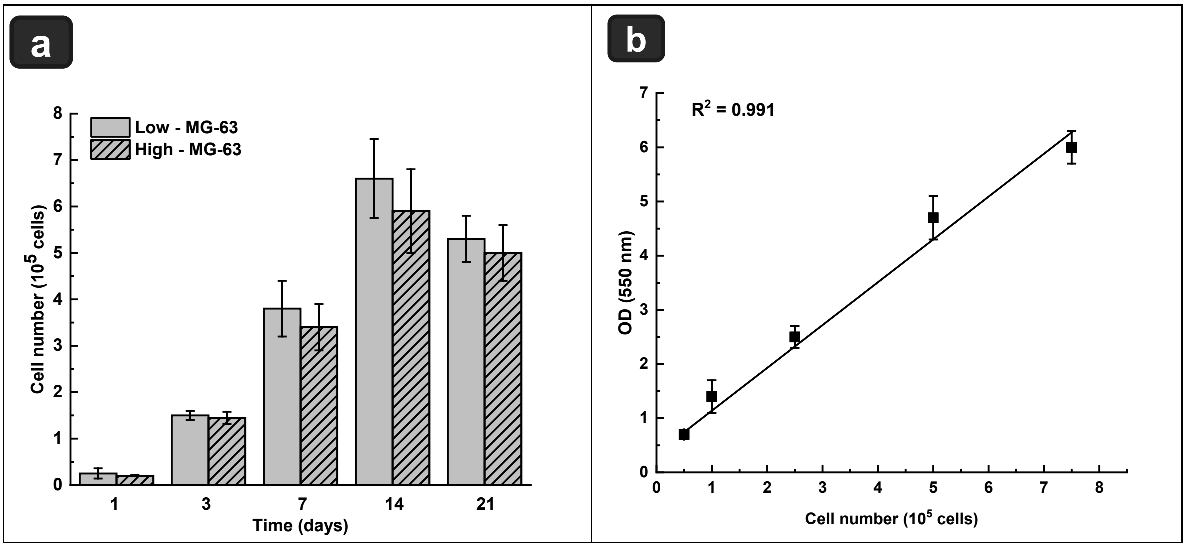

Figure S5. The growth curves of MG-63 cells on scaffolds (a). The control group and the test group were monitored by MTT assay (b). Each point represents the average absorbance readings of three scaffolds.

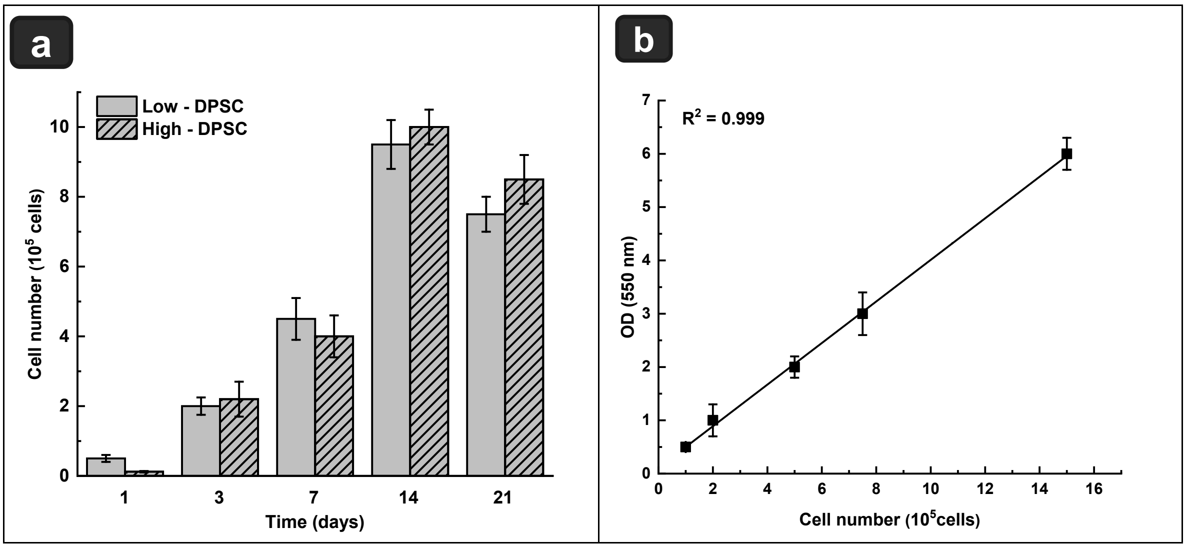

Figure S6. The growth curves of DPSC cells that were grown on scaffolds (a). The control group and the test group were monitored by MTT assay (b). Each point represents the average absorbance readings of three scaffolds.

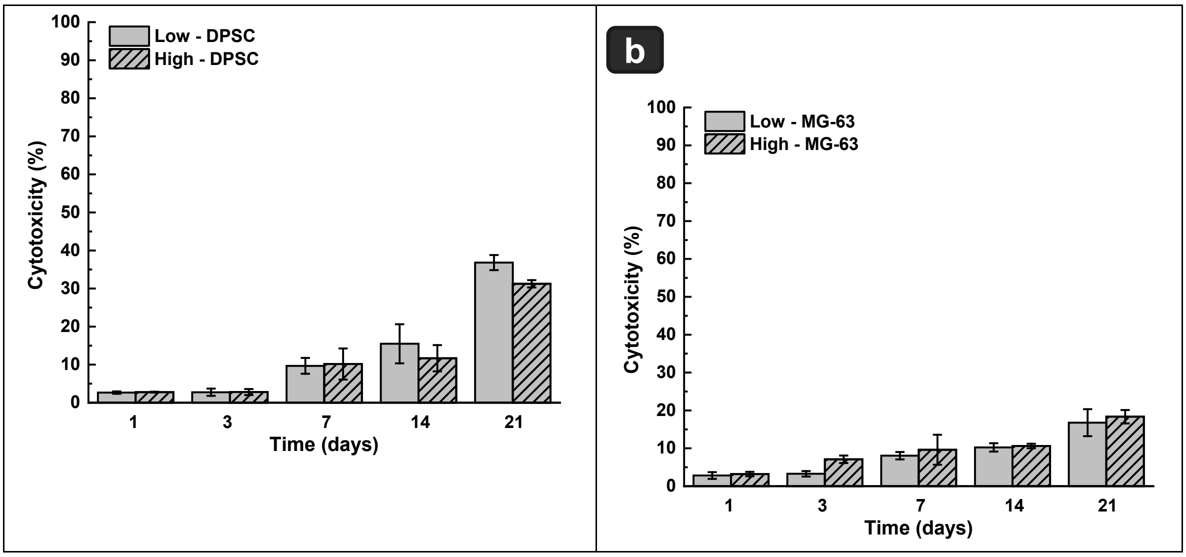

Figure S7. Cytotoxicity assay of LDH release from DPSCs (a) and MG-63 cells (b) grown on hydrogels.
